# The hidden impact of alcohol on young victims: an analysis of police-related alcohol offences resulting in hospitalisation

**DOI:** 10.23889/ijpds.v9i5.2593

**Published:** 2024-09-18

**Authors:** Scott Sims

**Affiliations:** 1 University of British Columbia

## Background

Alcohol-related harm (ARH) remains a significant public health concern, particularly affecting young individuals who are disproportionately impacted. This study focuses on the often-overlooked impact of alcohol on young victims involved in alcohol-related police incidents resulting in hospitalisation. While existing research often centres on the drinker, this study explores the characteristics of both offenders and victims, analysing offence types and investigating the under-ascertainment of ARH in hospital records.

## Methods

Our research, conducted as a retrospective longitudinal study in Western Australia, utilises linked data from hospital admissions, emergency department (ED) presentations, and police incident records. The study includes 22,747 individuals aged 12–24 involved in alcohol-related police incidents between 2004 and 2015.

## Results

Female victims were 10 times more likely than males to be involved in alcohol-related police incidents, while women had a 2 times higher likelihood of undiagnosed alcohol-related hospital admissions than men. Aboriginal people were overrepresented as both offenders and victims. ED presentations and hospital admissions were prevalent, with head injuries being the most common outcome. Notably, 71% of victims hospitalised did not receive an ARH diagnosis.

## Conclusions

A noticeable gap exists between hospital admissions associated with alcohol-related incidents and cases officially classified as ARH in the hospital system. This difference points to a more significant issue of substantial under-ascertainment or insufficient identification of ARH than previously acknowledged. Our study emphasises the need for targeted interventions, collaborative efforts between health and justice agencies, and ongoing research to address the hidden impact of alcohol on young victims.

